# Dual S-100-AE1/3 Immunohistochemistry to Detect Perineural Invasion in Nonmelanoma Skin Cancers

**DOI:** 10.1155/2015/620235

**Published:** 2015-01-18

**Authors:** Alma C. Berlingeri-Ramos, Claire J. Detweiler, Richard F. Wagner, Brent C. Kelly

**Affiliations:** ^1^Department of Dermatology, University of Texas Medical Branch, 301 University Drive, Galveston, TX 77555-5302, USA; ^2^Department of Pathology, Duke University Medical Center, Durham, NC 27710, USA

## Abstract

*Background*. Perineural invasion (PNI) is an adverse prognostic histologic finding and increases the risk of local recurrence and metastasis. *Objective*. We aimed to determine if dual immunohistochemical (IHC) staining with S-100 and AE1/3 would increase the detection of PNI on nonmelanoma skin cancers (NMSCs). *Methods*. We collected 45 specimens of NMSCs in which there was clinical suspicion for PNI. Two dermatopathologists independently reviewed the tumors for the unequivocal presence of PNI. *Results*. Unequivocal PNI was present on 10 of the 45 tumors by H&E staining and on 15 of the 45 tumors by IHC staining. Large nerves (>0.1 mm) were involved in 3 of 10 H&E-stained cases and 3 of 15 IHC-stained cases, with 2 of the 4 cases demonstrating large nerve involvement with both staining methods. Of the 8 cases of PNI detected only on IHC, 7 were small nerves (≤0.1 mm). *Limitations*. All cases were selected because they were clinically suspicious for PNI, and this may be considered selection bias. *Conclusions*. PNI detection may be increased using dual S-100 and AE1/3 staining, but the majority of additional cases detected were small nerves. The clinical significance, given the small size of the involved nerves, is unclear.

## 1. Introduction

Perineural invasion (PNI) is growth of a tumor within the perineural space of a nerve or within the nerve fascicles [1–3]. PNI, particularly for cutaneous squamous cell carcinoma (cSCC), is considered an adverse prognostic histologic finding and increases the risk of local recurrence and metastasis [4, 5]. The current edition of the American Joint Committee on Cancer's Staging Manual includes PNI as a high risk feature for nonmelanoma skin cancers (NMSCs) because of its predictive value for aggressive behavior [6].

We aimed to determine if dual immunohistochemical (IHC) staining with both S-100 and AE1/3 would increase the detection of PNI on NMSCs on pathohistology.

## 2. Materials and Methods

Our electronic dermatopathology records were reviewed using an electronic SNOMED retrieval system for two types of NMSCs, basal cell carcinomas (BCCs) and squamous cell carcinomas (SCCs), in which the clinical information included the term “perineural” from June 2003 to June 2008. These records were evaluated and all cases (*n* = 45) of NMSC in which there was a clinical concern for PNI were included. The clinical information was reviewed and, in most cases, the clinician was concerned about PNI because of size of the tumor, pain, or suspicion based on Mohs frozen sections.

Tissue sections were cut at 3–5 microns and mounted on positively charged slides. Prior to treatment, all sections were dried in a slide oven at 60 degrees for 30 minutes to ensure that the sections adhered to the slides and were completely dry. Sections were deparaffinized in 4 changes of xylene for 5 minutes each and then rehydrated through a series of graded alcohols with a final rinse in distilled water. Endogenous peroxides were quenched by soaking sections in two changes of 3% methanol H_2_O_2_ for 5 minutes each.

Prior to IHC, sections were treated with antigen retrieval to facilitate antibody binding to antigen. Briefly, slides were incubated in the Black and Decker Vegetable Steamer for 20 minutes in Target Retrieval Solution (Dako Corporation, Carpinteria, CA; Cat. # S1699) preheated to 99°C. They were then taken out of the steamer and placed on an open counter to cool for 20 minutes in the solution. The slides were then rinsed in 3 changes of distilled water and placed into a container of tris-buffered saline (Signet Pathology Systems, Inc., Dedham, MA; Cat. # 2380) for 5 minutes to decrease the surface tension of the slides and facilitate coating by the following reagents.

Slides were loaded horizontally onto the Dako Autostainer where each of the following steps was automated. Tris-buffered saline was used to rinse the sections between each of the IHC steps. Both avidin and biotin from the Avidin Biotin Blocking Kit (Vector Laboratories, Burlingame, CA; Cat. # SP2001) were diluted in Antibody Diluent (Dako) at a ratio of 1 mL avidin or biotin to 5 mL diluent.

Diluted avidin was applied to sections and incubated for 7 minutes. AE1/3 (Signet number 464-01) at a concentration of 1 : 200 was incubated for 30 minutes. Sections were then incubated in LSAB2, universal secondary antibody (Dako) for 15 minutes, followed by LSAB2, label (Dako) for 15 minutes; then chromogen liquid DAB (Dako) was applied for 5 minutes. The slides were then run again in the autostainer using the primary antibody S-100 (Dako number Z0311) at a concentration of 1 : 4000 for 30 minutes. The tertiary reagent used here was a streptavidin-alkaline phosphatase conjugate (Millipore # 20841). Fast red was used as the chromogen. The slides were then taken off the autostainer and rinsed in distilled water, manually counterstained with Harris Hematoxylin (Fisher Scientific) for 1 minute, rinsed in distilled water followed by 0.25% ammonia water to blue and then a final rinse in distilled water. They were then dehydrated through graded series of alcohols and cleared in four changes of xylene and coverslipped with synthetic glass and Permount mounting media.

Dual IHC staining showed red chromogen staining of peripheral nerve nuclei and cytoplasm with S-100 and brown cytoplasmic staining of epithelial cells with AE1/3.

Two dermatopathologists independently reviewed the H&E sections and IHC sections for the unequivocal presence of PNI. PNI was defined as malignant appearing epithelial cells surrounding more than 50% of the nerve and tumor present within the perineurium or within the nerve fascicles [1–3]. Large nerves were defined as >0.1 mm, and small nerves were defined as ≤0.1 mm [1].

## 3. Results

**Figure 1:**
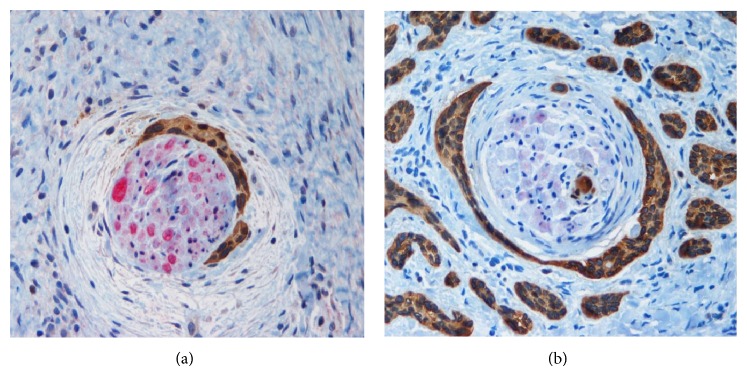
Perineural invasion of squamous cell carcinoma (brown) inside the perineurium of the nerve (red). (a) IHC with dual staining for S-100 and AE1/3, original magnification ×200. Squamous cell carcinoma (brown) surrounding nerve, focally penetrating perineurium, and within fascicles. (b) IHC with dual staining for S-100 and AE1/3, original magnification ×200. IHC: immunohistochemistry.

Table 1 presents the findings for the 45 NMSCs (17 BCCs and 28 SCCs) studied. Examples of dual staining results are seen in Figure 1.

**Figure 2:**
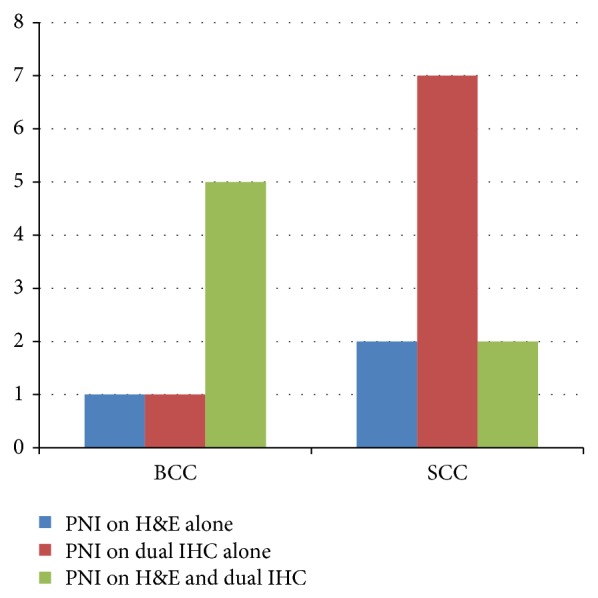
Comparative analysis between routine H&E examination and dual IHC using antibodies for S-100 and AE1/3 in the evaluation of PNI in cases of BCC and SCC. PNI: perineural invasion; H&E: hematoxylin and eosin; IHC: immunohistochemistry; BCC: basal cell carcinoma; SCC: squamous cell carcinoma.

Overall, 18 cases (40%; 7 BCCs and 11 SCCs) had evidence of PNI on either routine examination or dual IHC staining. In the majority of cases (34 cases, 75.6%) the findings on H&E and dual IHC-stained tissue sections correlated with the presence or absence of PNI. This included 27 cases (60%) in which PNI was absent via both methods and 7 cases (15.6%) in which PNI was identified using both methods. In 8 cases (44.4%; 1 BCC and 7 SCCs) dual IHC was the only method by which PNI was demonstrated compared to 3 cases (16.7%; 1 BCC and 2 SCCs) in which H&E alone yielded positive findings (Figure 2).

Of these 3 cases identified via H&E alone, 1 corresponding IHC sample was inadequate due to loss of tissue. PNI on a second case was not found on dual IHC staining because IHC staining revealed that the tumor surrounded the nerve without invasion of perineurium.

**Figure 3:**
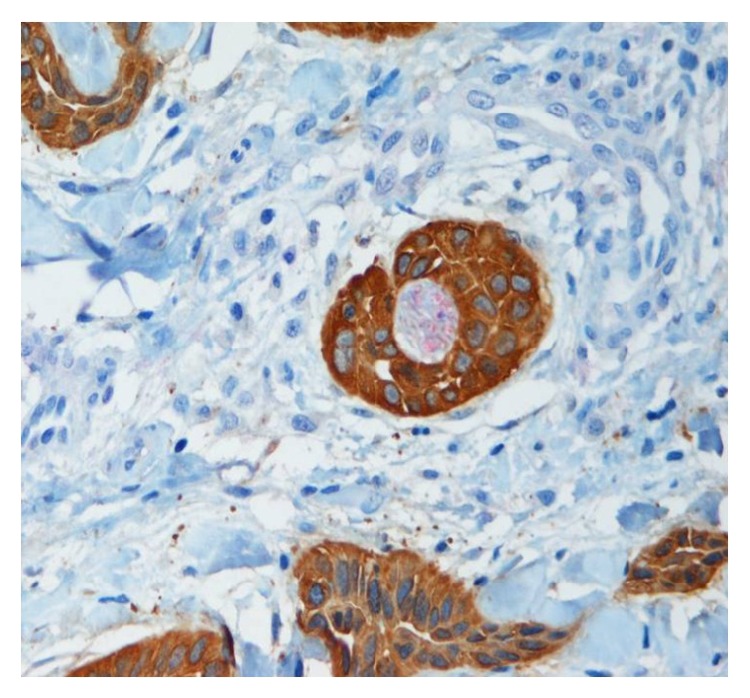
Perineural invasion. A small nerve branch (red) completely surrounded by squamous cell carcinoma (brown) in a case in which perineural invasion was not detected on H&E. IHC with dual staining for S-100 and AE1/3, original magnification ×200. H&E: hematoxylin and eosin; IHC: immunohistochemistry.

Of the 8 cases in which PNI was found on dual IHC staining only, 7 cases (87.5%) involved small nerves (≤0.1 mm) (Figure 3). Seven of 8 (87.5%) were SCC cases, and 1 of 8 (12.5%) was a BCC case. Large nerves (>0.1 mm) were identified in 3 of 10 (30%) of H&E-stained cases and 3 of 15 (20%) of IHC-stained cases, with 2 of the 4 large nerve cases identified with both types of staining.

Out of the 7 cases in which PNI was identified via both methods there was 1 case in which H&E staining identified PNI in a large nerve, but IHC staining identified PNI in a small nerve. In the remaining 6 cases the diameter of the involved nerve found on H&E examination remained in the same nerve size category following IHC staining.

There were 0 cases in which the dermatopathologists differed in diagnosis of presence or absence of PNI.

## 4. Discussion

PNI in NMSC, particularly (cSCC), is considered to carry a more aggressive prognosis with increased rates of recurrence and metastasis [4, 5]. In a study by Rowe et al. [5] SCC of the skin, ear, and lip with histologic PNI had a local recurrence rate of 47.2% and a metastatic rate of 47.3%. In a study by Goepfert et al. [4] patients with cSCC with PNI had regional lymph node metastases (35% of patients) and distant metastases (15% of patients) significantly more frequently than in those without PNI.

Incidence of PNI varies greatly between studies depending on many potential variables, including number of histological sections examined, stains used, and diagnostic criteria. Leibovitch et al. [7, 8] found PNI in 283 of 10,035 basal cell carcinomas (BCCs) (2.74%) and in 70 of 1177 SCCs (5.95%) that were treated with Mohs surgery between 1993 and 2002. PNI is seen more commonly in SCCs than BCCs, and patients with SCC with PNI have a worse prognosis than those with BCC with PNI [7–9]. PNI is also more common in recurrent rather than previously untreated malignancies, and prognosis is worse for those with PNI in a recurrence [2, 4, 9]. Prognosis is also worse in patients with extensive rather than local PNI [9].

PNI is separated into clinical and incidental types. Clinical PNI describes patients with clinical symptoms or signs, such as cranial neuropathy and/or radiographic evidence of tumor involvement of a nerve [1, 10]. The most common symptom of PNI is paresthesias such as formication which can subsequently develop into feelings of pain, numbness, and/or weakness [10]. The trigeminal (CN V) and facial (CN VII) nerves are the most commonly involved [2, 4]. Imaging evidence of PNI is associated with a poorer prognosis [11]. Even with clinical symptoms of PNI, diagnosis can be delayed for more than 6 months to a year if suspicion is low [2]. Incidental PNI describes asymptomatic patients without radiographic evidence but with histopathologic evidence of PNI [1, 10]. Approximately 30% to 40% of patients with PNI have clinical evidence while for the majority of cases it is an incidental finding [2]. Patients with clinical PNI have a worse prognosis than those with incidental PNI [2, 10].

Detection of PNI is routinely performed histologically with H&E staining, but accurate identification of PNI can be difficult. Histological evidence of PNI is the presence of malignant cells within the perineural space of a nerve or within the nerve fascicles [1–3]. However, there is currently no standard definition of PNI, and some studies have included tumor near or abutting the outer perineurium [12]. Perineural inflammation and nerve fiber degeneration can be used as clues to nearby PNI [13]. Tumor cells can be hard to distinguish from normal perineurium and stroma [12]. Tumor cells in the perineural space may appear histologically different from the tumor from which they originate and may only show subtle signs of atypia [13]. There are at least four benign mimics of PNI that need to be considered and excluded when diagnosing PNI: peritumoral fibrosis, reexcision PNI, reparative PNI, and epithelial sheath neuroma [14]. Some studies say that perineural spread can have skip areas, where PNI is not visible on adjacent sections of tissue [12]. Other studies do not support the idea of skip areas. For example, Panizza et al. [15] did not find skip lesions in their study and suspected that skip areas are really a processing artifact. IHC staining for cSCC has been studied by Kelso et al. [16] who applied S-100 and p75^NGFR^ IHC staining to cSCC cases and found increased detection of PNI with the use of each. Standardized pathology protocols stringently defining PNI as tumor cells within the perineural space or between fascicles, staining of ambiguous cases, and documentation of the size of involved nerves could help increase the accuracy of PNI diagnosis and estimation of prognosis [1–3, 12].

Another factor to consider regarding PNI is the size of the nerve involved. It is currently unclear whether the size of the nerve involved plays a prognostic role in PNI. One variable contributing to this is the ambiguity of the words “large nerve” and “small nerve.” Some studies define a large nerve as >0.1 mm, while others define it as ≥0.1 mm. There have been calls to officially define small nerves as ≤0.1 mm and large nerves as >0.1 mm, thus placing 0.1 mm nerves into the small nerve group [1]. Ross et al. [12] demonstrated that, in patients with SCC, PNI of large-caliber nerves (≥0.1 mm) had worse outcomes than PNI of small-caliber nerves (<0.1 mm). McCord et al. [17] also found a lower rate of local control with involvement of a named nerve pathway, though nerve diameter was not specifically defined. Carter et al. [18] demonstrated increased risk of nodal metastasis and death from disease with large nerve (≥0.1 mm) involvement. On the other hand, Lin et al. [9] demonstrated no difference in outcome in relation to the size of the involved nerve in patients with SCC or BCC when defining large nerves as >0.1 mm. Many studies have shown that large nerve PNI is associated with other risk factors such as recurrent tumor, larger tumor diameter, greater maximum depth of tumor, invasion beyond the subcutaneous fat, multiple nerve involvement, infiltrative growth, and lymphovascular invasion [12, 18]. It has been postulated that it is these associated risk factors rather than large nerve PNI that have resulted in poorer outcomes in patients [18]. Further studies are needed to truly determine significance, and universal guidelines for labeling of nerve sizes are needed for this area of research to progress.

There are no universal guidelines for the treatment of NMSC with PNI and current recommendations do not use PNI nerve size in the decision of treatment method [12]. Most are treated with radiotherapy with or without surgical excision via standard or Mohs micrographic surgery [1, 17, 19]. Mendenhall et al. [2] recommended treating NMSC with PNI with resection and postoperative radiotherapy if the cancer is resectable and treating with definitive radiotherapy for unresectable cases without consideration for PNI nerve size either way. Ross et al. [12] suggested adjuvant radiotherapy only when PNI involves large nerves greater than or equal to 0.1 mm. Currently, there are no clear evidence-based treatment guidelines, and physicians and patients are faced with choosing between surgery, radiotherapy, or both.

The 7th edition of the American Joint Committee on Cancer's Cancer Staging Manual (AJCC-7) for cSCC includes perineural invasion as a high risk feature, and the presence of two or more high risk features at any tumor size increases the stage from T1 to T2 [20]. Other high risk features include poorly differentiated or undifferentiated tumors, primary anatomical site on the ear or the nonhair bearing lip, thickness greater than 2 mm, and Clark level of at least IV [20]. There has been a proposal by Jambusaria-Pahlajani et al. [21] to classify the presence of one high risk factor as stage T2a, two to three high risk factors as stage T2b, and four high risk factors as stage T3. The results of their study showed worse outcomes (nodal metastases and death from cSCC) in T2b tumors compared to T2a tumors [21]. In a study by Schmitt et al. [22] it was shown that T2 tumors larger than 2 cm in the AJCC-7 criteria and T2b tumors in the alternative criteria by Jambusaria-Phlajani et al. have positive sentinel lymph node biopsy (SLNB) rates over 10% and thus warrant SLNB.

We simultaneously utilized the unique staining qualities of two different antibodies for the purpose of detection of PNI in NMSCs. In this pilot study, we did increase our detection of PNI compared to H&E alone. Dual IHC analysis using antibodies to S-100 and AE1/3 enhanced detection of suspicious foci by providing distinct staining patterns of peripheral nerves and malignant appearing epithelial cells, respectively. Dual IHC staining was particularly useful to resolve the diagnostic dilemma of perineurial proliferation and peritumoral fibrosis. The majority of these additional detections were in SCC cases (87.5%) and involved small nerves (87.5%). However, it is unclear if increased detection of small involved nerves would have clinical significance [9, 12]. All cases were selected because they were clinically suspicious for PNI, and this may be considered a form of selection bias and thus a pitfall of the study.

In cases with PNI, dual IHC did not significantly alter nerve diameter measurements. Dual IHC staining also increases the cost of diagnosis. Thus, additional dual IHC staining may not be warranted in cases in which PNI is clearly identified on initial H&E sections.

Dual IHC staining with S-100 and AE1/3 may be useful in cases where PNI is suspected but unclear on H&E analysis, especially in the case of peritumoral fibrosis and proliferation of perineurium.

## Figures and Tables

**Table 1:** Detection of PNI on H&E and dual IHC staining.

Case number	Tumor type	PNI H&E	Nerve size (mm)	PNI dual IHC	Nerve size (mm)	Notes
1	BCC	−		−		
2	BCC	+	0.01	+	0.04	
3	BCC	−		−		
4	BCC	−		−		
5	BCC	+	0.06	−		
6	BCC	−		−		
7	BCC	−		−		
8	BCC	+	0.08	+	0.06	
9	BCC	+	0.11	+	0.08	
10	BCC	−		−		
11	BCC	−		−		
12	BCC	+	0.14	+	0.15	
13	BCC	−		−		
14	BCC	−		−		
15	BCC	+	0.07	+	0.07	
16	BCC	−		+	0.02	
17	BCC	−		−		
18	SCC	+	0.18	+	0.11	
19	SCC	−		+	0.03	
20	SCC	+	0.04	−		IHC staining more clearly demonstrated that tumor did not invade perineurium
21	SCC	−		−		
22	SCC	−		−		
23	SCC	−		−		
24	SCC	−		−		
25	SCC	−		−		
26	SCC	−		−		
27	SCC	−		−		
28	SCC	−		−		
29	SCC	−		−		
30	SCC	−		−		
31	SCC	−		−		
32	SCC	−		+	0.03	
33	SCC	−		+	0.03	
34	SCC	−		−		
35	SCC	+	0.06	−		Tissue block was cut through on sectioning for IHC
36	SCC	−		−		
37	SCC	−		+	0.06	
38	SCC	−		+	0.04	
39	SCC	+	0.05	+	0.02	
40	SCC	−		−		
41	SCC	−		−		
42	SCC	−		+	0.05	
43	SCC	−		+	0.12	
44	SCC	−		−		
45	SCC	−		−		

PNI: perineural invasion; H&E: hematoxylin and eosin; IHC: immunohistochemistry; BCC: basal cell carcinoma; SCC: squamous cell carcinoma.
